# Head-to-head comparison of the RMI and ADNEX models to estimate the risk of ovarian malignancy: a systematic review and meta-analysis of external validation studies

**DOI:** 10.1136/bmjopen-2025-104141

**Published:** 2025-10-07

**Authors:** Lasai Barreñada, Ashleigh Ledger, Agnieszka Kotlarz, Paula Dhiman, Gary Stephen Collins, Laure Wynants, Jan Yvan Jos Verbakel, Lil Valentin, Dirk Timmerman, Ben Van Calster

**Affiliations:** 1 Department of Development and Regeneration, KU Leuven, Leuven, Belgium; 2 Department of Gynecology and Oncology, Faculty of Medicine, Jagiellonian University Medical College, Kraków, Poland; 3 Centre for Statistics in Medicine, Nuffield, Department of Orthopaedics, Rheumatology and Musculoskeletal Sciences, University of Oxford, Oxford, UK; 4 Department of Epidemiology, CAPHRI Care and Public Health Research Institute, Maastricht University, Maastricht, Netherlands; 5 Department of Public Health and Primary Care, KU Leuven, Leuven, Belgium; 6 Leuven Unit for Health Technology Assessment Research (LUHTAR), KU Leuven, Leuven, Belgium; 7 Department of Obstetrics and Gynaecology, Skåne University Hospital, Malmö, Sweden; 8 Department of Clinical Sciences Malmö, Lund University, Lund, Sweden; 9 Department of Obstetrics and Gynaecology, University Hospitals Leuven, Leuven, Belgium

**Keywords:** Meta-Analysis, Gynaecological oncology, STATISTICS & RESEARCH METHODS

## Abstract

**Abstract:**

**Objectives:**

Assessment of Different NEoplasias in the adneXa (ADNEX) and Risk of Malignancy Index (RMI) are models that estimate the risk of malignancy in ovarian masses based on clinical and ultrasound information. The aim is to perform a meta-analysis of studies that compared the performance of the two models in the same patients (‘head-to-head comparison’).

**Design:**

Systematic review and meta-analysis.

**Data sources:**

Systematic literature search from publication of ADNEX model (15/10/2014) up to 31/07/2024 in Embase, Web of Science, Scopus, Medline (via PubMed) and EuropePMC.

**Eligibility criteria for selecting studies:**

We included all studies that externally validated the performance of ADNEX (with or without CA125) and RMI on the same data.

**Data extraction and synthesis:**

Two independent reviewers extracted data using a standardised extraction sheet. We assessed risk of bias using PROBAST. We performed random effects meta-analysis of the area under the receiver operating characteristic curve (AUC), sensitivity, specificity and clinical utility (net benefit, relative utility and probability of being useful in a hypothetical new centre) at thresholds commonly used clinically (10% risk of malignancy for ADNEX, 200 for RMI).

**Results:**

We included 11 studies comprising 8271 tumours. Most studies were at high risk of bias. The summary AUC to distinguish benign from malignant tumours in operated patients for ADNEX with CA125 was 0.92 (95% CI 0.90 to 0.94) and for RMI it was 0.85 (0.81 to 0.89). Sensitivity and specificity for ADNEX with CA125 were 0.93 (0.90 to 0.96) and 0.77 (0.71 to 0.81) and for RMI, they were 0.61 (0.56 to 0.67) and 0.92 (0.89 to 0.94). The probability of the test being useful in a hypothetical new centre in operated patients was 96% for ADNEX with CA125 and 15% for RMI at the selected thresholds.

**Conclusions:**

ADNEX has better discrimination and clinical utility than RMI.

**STRENGTHS AND LIMITATIONS OF THIS STUDY**

- Comprehensive systematic review of all head-to-head external validation studies of Assessment of Different NEoplasias in the adneXa (ADNEX) and Risk of Malignancy Index (RMI).
- Risk of bias assessment of included studies based on the PROBAST tool.
- High risk of bias in most studies might have affected the results of included studies and so of our meta-analysis.
- Model calibration was not assessed due to lack of reporting in most studies.

## Background

 To choose the optimal management of an ovarian mass, the characterisation of the mass as benign or malignant is essential to improve treatment outcomes.^1 2^ If the mass is likely to be malignant, treatment in a referral centre for gynaecological oncology improves the outcome.^3 4^ The Assessment of Different NEoplasias in the adneXa (ADNEX) model^5^ and the Risk of Malignancy Index (RMI)^6^ have become key tools for estimating the risk of an ovarian mass being malignant.

RMI provides a non-negative value that is associated with malignancy.^6^ A commonly used cut-off to classify a tumour as high risk for malignancy is 200,^7^,^11^ but other cut-offs have also been suggested.^7 12 13^ RMI is based on the CA125 serum level (U/ml), menopausal status and the presence of five ultrasound features. The model was published in 1990, based on data from 143 patients from a single hospital, of whom 42 had a malignant tumour. Modifications of the RMI have been published (RMI 2, RMI 3 and RMI 4^14^,^16^), but in several countries, the use of the original RMI is recommended.^7^,^9^ ADNEX estimates the risk that an ovarian tumour is benign, borderline, stage I primary invasive, stage II-IV primary invasive or a metastasis in the ovary from another primary tumour.^5^ The estimated risk of malignancy is calculated as the sum of the risks of the four malignant subtypes. ADNEX uses nine predictors: patient’s age (years), maximum diameter of lesion (mm), proportion of solid tissue (calculated as the maximum diameter of the largest solid component divided by the maximum diameter of the lesion), number of papillary projections (0, 1, 2, 3, or >3), presence of acoustic shadows, presence of ascites, presence of more than 10 cyst locules, serum CA125 (U/ml) and type of centre (oncology centre vs other). There is also an ADNEX version without serum CA125. ADNEX was published in 2014 based on data from 5909 patients. A commonly used cut-off, recommended in an international consensus statement, is a risk of malignancy of 10%.^1^ To the best of our knowledge, there are no published systematic reviews with meta-analysis that focus on a head-to-head comparison of ADNEX with RMI, that is, a comparison of studies that assess performance of both models on the same data. However, one systematic review of models for women with suspected ovarian cancer added a limited subanalysis to compare sensitivity and specificity at the 10% risk of malignancy cut-off for ADNEX with CA125 (96% and 67%, respectively) and at the 200 cut-off for RMI (66% and 89%, respectively) based on one published study and one unpublished study.^17^ In some national guidelines, RMI is still recommended for triaging patients with ovarian tumours for referral to an oncology centre.^7^,^1113^ Therefore, an extended head-to-head comparison of the performance of RMI and ADNEX is needed.

This is an extension of a recently published systematic review and meta-analysis of external validations of the ADNEX model.^18^ The aim is to present a systematic review and meta-analysis of studies that externally validated ADNEX and the original RMI (RMI 1) on the same patients and to compare the performance of the two models.

## Methods

### Protocol registration

We report this study according to the Preferred Reporting Items for Systematic Reviews and Meta-Analysis (PRISMA) and TRIPOD-SRMA checklist.^19 20^ The study protocol was prospectively registered in the international prospective register of systematic reviews (PROSPERO; ID CRD42023449454).

### Eligibility criteria

This is a follow-up study of a systematic review of ADNEX.^18^ Inclusion and exclusion criteria are the same as in the systematic review of ADNEX, with the restriction that eligible studies also had to present metrics for the original RMI at the 200 cut-off for the same study population. The 200 cut-off was selected as it is a cut-off often recommended for referring a patient to an oncological centre.^7^,^11^

The inclusion criteria were as follows: any publication that externally validates the performance of the ADNEX model and RMI simultaneously on the same study population using any study design and any study population consisting of patients with an adnexal mass.

The exclusion criteria were as follows: (1) studies that do not evaluate the predictive performance (in any way) of ADNEX and RMI simultaneously in the same population, (2) studies that only evaluate the predictive performance of updated versions of ADNEX or RMI. Updating can refer to recalibration, refitting, extension with additional predictors or any type of modification to the original formula. Thus, RMI 2, 3 or 4 or any updated ADNEX model is not included in this review. (3) Studies for which only an abstract is available, either because we could not get access to the full text, or because no full text exists (eg,conference abstracts) and (4) case studies presenting the performance in individual patients. When the full text was not accessible, we tried to obtain it by contacting the corresponding authors via email.

### Information sources and search strategy

The search was conducted on the 31 July 2024. The search string and overall search strategy were created with the help of biomedical librarians at the KU Leuven Libraries. Embase, Web of Science, Scopus and Medline (via PubMed) were searched for published studies, and EuropePMC for preprints. The databases were searched from the publication of the first ADNEX article (15/10/2014) until 31/07/2024. The full search strategy is provided in online supplemental material S1.

### Study selection

The studies identified in our search were imported into Zotero reference manager, where they were automatically deduplicated. The deduplicated records were then imported into the Rayyan web application for further manual deduplication by LB and subsequent screening of the title and abstract by two independent authors (LB and AL). Disagreements in eligibility were resolved by discussion between LB and AL.

Three of the authors (BVC, LV and DT) were members of the IOTA group that developed ADNEX. Therefore, we divided the studies into those that were linked or not linked to IOTA. A study was linked to IOTA if it was co-authored by a member of the IOTA steering committee (iotaplus.org/en/research/iota-models). IOTA-linked papers, as well as a few others with a potential conflict of interest (ie, including authors that are or were IOTA collaborators), were assessed by two authors who are independent from IOTA (PD and GSC, medical statisticians with expertise in prediction modelling). All other studies were independently assessed by LB and AL. Disagreements were resolved by discussion between reviewers (pair of LB-AL or GSC-PD), and for the non-IOTA papers, by discussion with authors BVC, LV and JYJV.

### Data extraction and data items

Information was collected and entered into a standardised data extraction form in Microsoft Excel by LB, AL, PD and GSC. The extraction process focused on study design, target population, reference standards, sample size, performance results, completeness of reporting, quality of methodology and risk of bias (online supplemental Table S1). The extraction form was structured based on CHARMS (Checklist for critical Appraisal and data extraction for systematic reviews of prediction Modelling Studies), TRIPOD (Transparent Reporting of a multivariable prediction model for Individual Prognosis Or Diagnosis (online supplemental Table S2) and PROBAST (Prediction model Risk of Bias Assessment Tool) tools.^21^,^23^

To describe the performance of ADNEX, we collected information regarding discrimination (AUC), calibration (calibration slope, calibration intercept, calibration plot), clinical utility (net benefit) and classification performance (sensitivity, specificity) at the 10% risk of malignancy threshold. We also collected information on the AUC, sensitivity and specificity at the 200 cut-off for RMI. When a performance measure was missing or the cut-off was not clear, we contacted the authors for clarification.

For each study, we evaluated the reporting of all relevant TRIPOD items for external validation studies. We also applied the PROBAST signalling questions and assessed the risk of bias within each domain (participants, predictors, outcome and analysis), as well as the overall risk of bias. The overall risk of bias is determined by the worst risk of bias rating across the domains. We included an explanation for our classification of the risk of bias. We critically appraised ADNEX and RMI independently because their predictors differ, the development information differs and the relevant performance measures differ. However, most signalling questions of PROBAST and most TRIPOD items are assessed at study level and are therefore equal for the two models.

### Statistical analysis and quantitative data synthesis

We performed a meta-analysis of centre specific results (or study specific when results for individual centres were not available) using random effects meta-analysis methods. We used a random effects model due to the anticipated between-study heterogeneity explained by different populations, settings or study designs.^24^ The AUC, sensitivity and specificity were meta-analysed on the logit scale. We calculated 95% CI for the summary estimates and estimated the between-study heterogeneity using τ squared and 95% prediction intervals (PI).^25 26^ Tau squared is the between-centre variance in the logit AUC, sensitivity or specificity, with higher values indicating more heterogeneity. The 95% prediction interval reflects the range of AUC, sensitivity and specificity we may expect in new centres: the wider, the larger the between-centre differences in performance. Unreported confidence intervals and prediction intervals were calculated based on Debray et al^24^ for AUC and with Wilson’s method for specificity and sensitivity.^24 27^

We express clinical utility to decide which patients to refer to an oncology centre as net benefit (NB), relative utility (RU) and P-useful.^28^,^32^ P-useful measures the probability that a model is useful in a new centre (NB difference with best default >0). The higher NB, RU and P-useful, the better. To evaluate clinical utility, we performed a trivariate meta-analysis of sensitivity, specificity and prevalence of malignancy for both ADNEX and RMI. We calculated utility under the assumption that we accept up to 10 referrals per referred malignancy. This is consistent with using a 10% risk of malignancy threshold. Since RMI does not provide risk estimates, we used a cut-off of 200. For NB and RU, we report 95% credible intervals (CrI) instead of CI. See online supplemental material S2 and S3 for more information on the methodology.

We compared ADNEX with CA125 with RMI and ADNEX without CA125 with RMI in two populations: (1) only operated patients, and (2) operated and conservatively managed patients combined. Thus, four meta-analyses were performed: (1) ADNEX with CA125 vs RMI in operated patients, (2) ADNEX without CA125 vs RMI in operated patients, (3) ADNEX with CA125 vs RMI in operated and conservatively managed patients combined and (4) ADNEX without CA125 vs RMI in operated and conservatively managed patients combined.

Reporting bias and small study effects were visually explored with funnel plots adapted for the AUC. The body of evidence was assessed with an adapted version of Grading of Recommendations Assessment, Development and Evaluation (GRADE).^33^ The analyses were performed in R V. 4.4.0 using package “metamisc” for AUC, “meta” and “mada” for sensitivity and specificity, and rjags for NB and RU. Bayesian methods for meta-analysis of clinical utility were computed using JAGS version 4.3.0.

### Patient and public involvement

Patients and members of the public were not involved in the design, conduct, reporting or dissemination plans of this research. This study is a systematic review and meta-analysis, which involves analysis of data from previously published studies and does not require direct patient or public involvement.

## Results

Thirteen studies presented results for both ADNEX and RMI (online supplemental Figure S1 and Table S3).^34^,^46^ Two studies were excluded from the systematic review and the meta-analysis because they validated only RMI 2.^43 46^

The characteristics of the included studies are summarised in table 1. The total number of patients included in the systematic review is 8271 with a median sample size of 326 patients per study (range 100–4905). The studies were conducted in 14 countries, and 11 validations of RMI and 15 validations of ADNEX, 11 for the version with CA125 and 4 for the version without CA125, were conducted. The complete extraction data and code to reproduce the results and figures are available in the OSF repository (https://osf.io/nt89z/).

**Table 1:** Characteristics of the eleven included studies

Study characteristics	N (%)	Comments
Unit of analysis		
Patient	10 (91)	
Tumour	1 (9)	
Region		
Asia	6 (55)	Includes two studies from Türkiye
Europe	4 (36)	
South America	1 (9)	
Number of centres		
1	7 (64)	
2–5	3 (27)	
>5	1 (9)	
Type of centre		
Oncology centre(s)	8 (73)	
Non-oncology centre(s)	0	
Both types of centres	1 (9)	
Unclear	2 (18)	
Type of study		
Prospective	4 (36)	
Retrospective	5 (45)	
Unclear	2 (18)	
ADNEX version		
With CA125	11 (100)	7 only used ADNEX with CA125, 4 used both
Without CA125	4 (36)	
Target population		
Surgically managed patients	10 (91)	
Surgically and non-surgically managed patients	1 (9)	

ADNEX, Assessment of Different NEoplasias in the adneXa.

### Critical appraisal: reporting and risk of bias

The 11 studies reported on average 19 out of 28 (68%) TRIPOD items for the validation of ADNEX, and 17.18 (61%) TRIPOD items for the validation of RMI (online supplemental Figure S2 and Table S4). The items that were reported in less than 35% of the validations both for ADNEX and RMI were justification of sample size (item 8) and justification of missing data management (item 9), specifying relevant measures of performance (item 10d) with confidence intervals (item 16) and comparing the participants’ characteristics in the original data with those in the validation data and discussing any differences (item 13 c).

**Figure 1:**
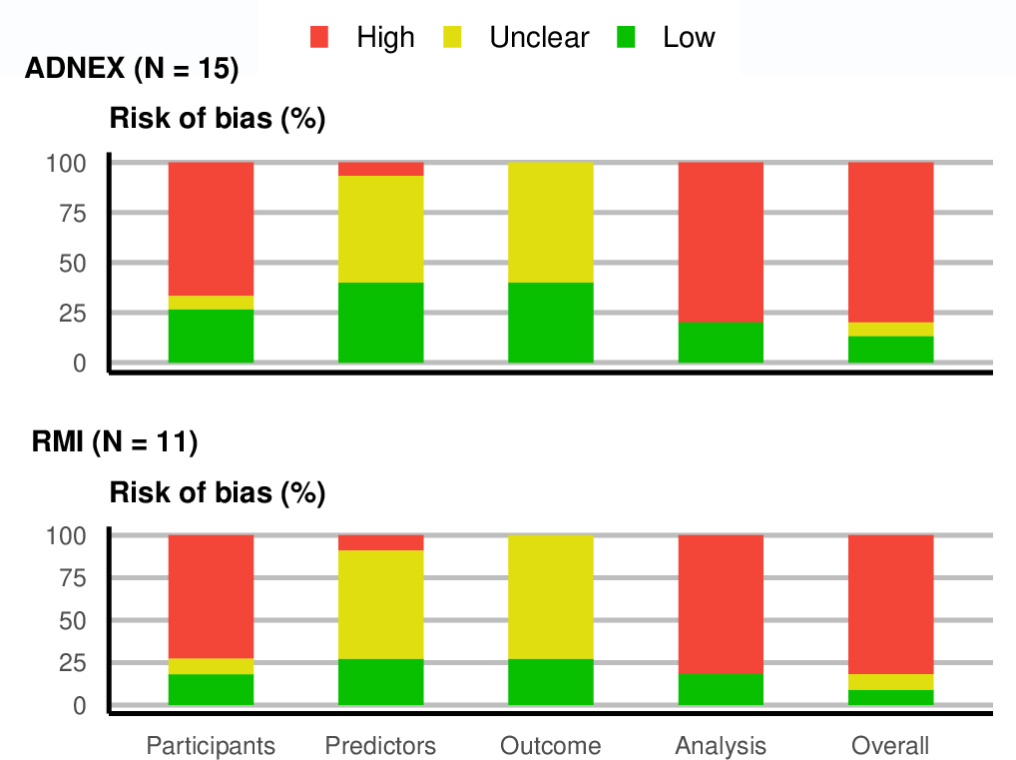
Risk of bias by subdomain and overall for Assessment of Different NEoplasias in the adneXa (ADNEX) and Risk of malignancy index (RMI) validations using the Prediction model Risk Of Bias ASsessment Tool (PROBAST). There are 15 validations of ADNEX and 11 validations of RMI.

Based on PROBAST, 12 of 15 validations (80%) of ADNEX were rated as overall high risk of bias, while 9 out of 11 validations (82%) of RMI were rated as overall high risk of bias mainly due to the small sample size and the exclusion of participants with missing data (figure 1 and online supplemental Figure S3 and S4). Most validations were unclear in the predictors domain due to not specifying when in relation to the inclusion scan serum levels of CA125 were measured, or for not reporting if the assessment of predictors was blinded to the outcome.

### Meta-analysis inclusions

One study was excluded from the meta-analysis because it only presented results stratified by the certainty of the ultrasound examiner when assessing the outcome,^41^ and another study was excluded for not reporting the cut-off used to calculate the sensitivity and specificity of ADNEX.^45^ Therefore, nine studies were eligible for meta-analysis.^34^,^4042 44^ Four studies presented results for both ADNEX versions (with and without CA125) and five presented results only for ADNEX with CA125. AUC results were reported in all studies except one.^40^ However, one study calculated AUC after applying the threshold (ie, based on a ROC curve with one single point) and thus was excluded from meta-analysis of AUC.^42^ Therefore, the meta-analysis of AUC was based on seven studies.^34^,^3944^ Sensitivity and specificity at a 10% risk of malignancy threshold for ADNEX were available (either in the publication or after contacting the authors) for all studies except one^37^ (online supplemental Table S4).

### Meta-analyses

**Figure 2:**
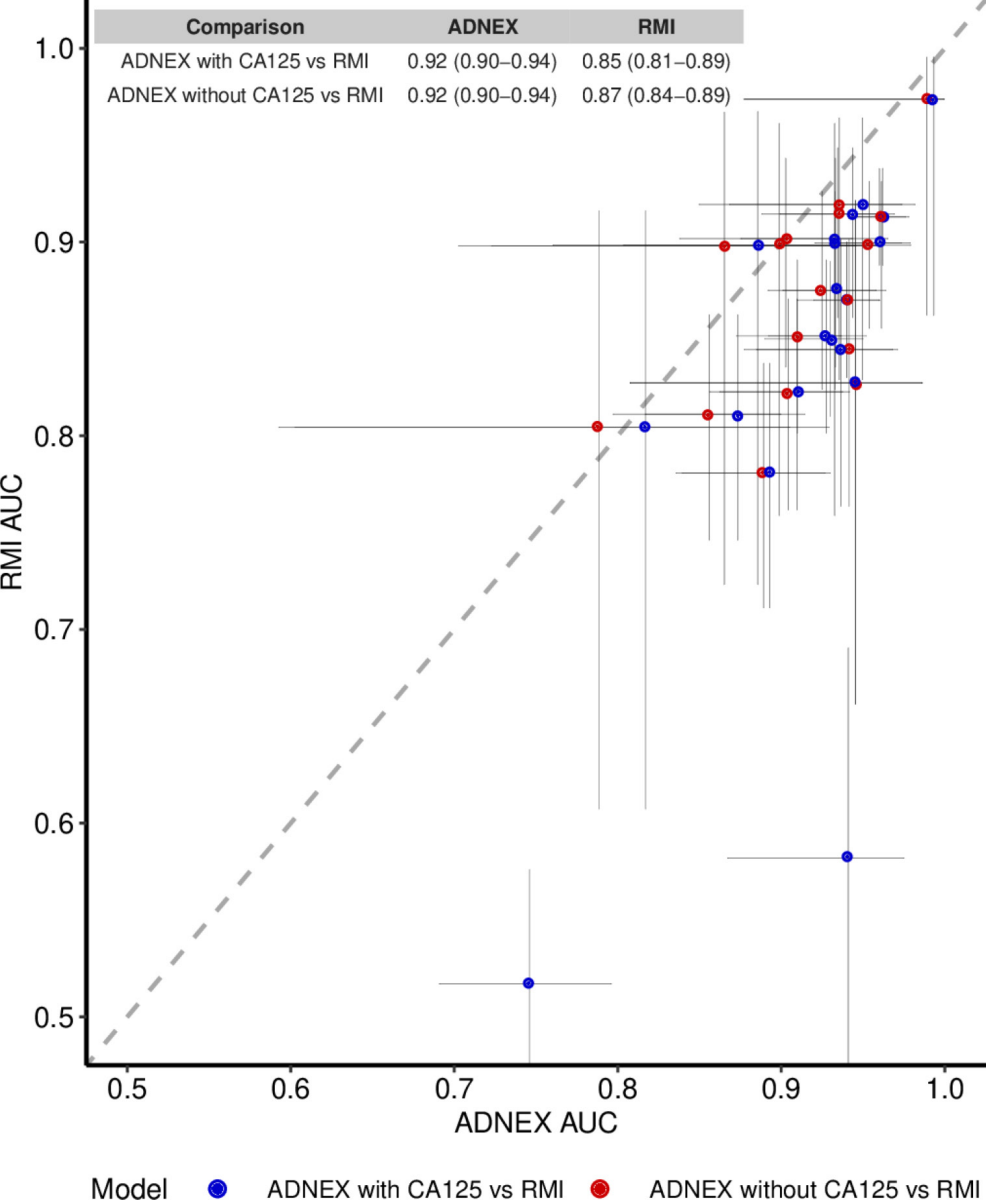
Comparison of the area under the receiver operating characteristic curve (AUC) of Assessment of Different NEoplasias in the adneXa (ADNEX) and (with and without CA125) and risk of malignancy index (RMI) per centre in the included studies for only operated patients. Vertical and horizontal lines present the CI of each centre’s AUC. Dots below the diagonal line indicate higher AUC for ADNEX than for RMI. Dots above the diagonal line indicate higher AUC for RMI.

Results of the meta-analysis of AUC are shown in table 2 and online supplemental Figure S5 and S6. Seven studies included in the meta-analysis compared the AUC of ADNEX (with CA125) with that of RMI in operated patients.^34^,^3944^ The summary estimate for the AUC in the four different meta-analyses varied between 0.92 and 0.94 for ADNEX, and between 0.85 and 0.89 for RMI. The summary estimate for AUC was higher for operated and conservatively managed patients combined (ADNEX 0.93–0.94; RMI 0.89) than for operated patients only (ADNEX 0.92; RMI 0.85–0.87). RMI had wider prediction intervals than ADNEX. Figure 2 shows the centre-specific or study-specific comparison of ADNEX with or without CA125 versus RMI in operated patients.

**Table 2:** Meta-analysis results for the area under the receiver operating characteristic curve (AUC)

Meta-analysis	Patients	Studies	Centres	Summary estimate	95% CI	95% PI	τ^2^
*Target population: operated patients*							
*For ADNEX with CA125*							
ADNEX	4668	7	26	0.92	0.90 to 0.94	0.82 to 0.99	0.30
RMI	4668	7	26	0.85	0.81 to 0.89	0.64 to 0.98	0.44
*For ADNEX without CA125*							
ADNEX	3773	4	22	0.92	0.90 to 0.94	0.85 to 0.97	0.16
RMI	3773	4	22	0.87	0.84 to 0.89	0.78 to 0.94	0.12
*Target population: Operated and conservatively managed patients*							
*For ADNEX with CA125*							
ADNEX	4905	1	17	0.93	0.91 to 0.96	0.85 to 0.99	0.27
RMI	4905	1	17	0.89	0.86 to 0.92	0.77 to 0.97	0.22
*For ADNEX without CA125*							
ADNEX	4905	1	17	0.94	0.93 to 0.96	0.88 to 0.99	0.26
RMI	4905	1	17	0.89	0.86 to 0.92	0.77 to 0.97	0.22

ADNEX, Assessment of Different NEoplasias in the adneXa; CI, confidence interval; PI, prediction interval; RMI, risk of malignancy index; t2, τ squared.

**Figure 3:**
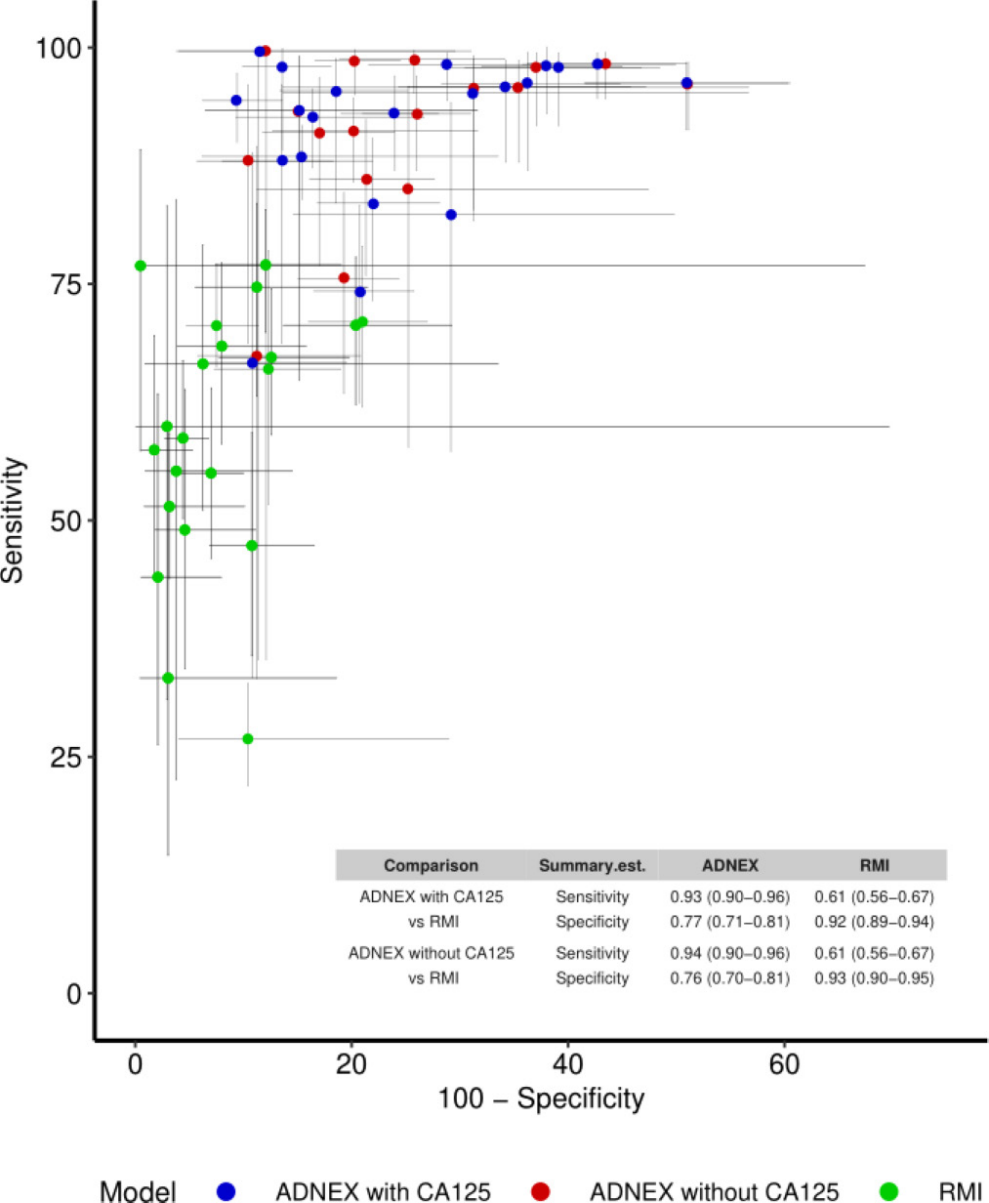
Sensitivity and specificity of Assessment of Different NEoplasias in the adneXa (ADNEX; 10% cut-off) and Risk of Malignancy Index (RMI; cut-off 200) in surgically managed patients illustrated in the space of a receiver operating characteristic curve. Each point represents the result of one study or centre and the lines represent the 95% CI.

The results of the meta-analysis for sensitivity and specificity are presented in table 3 and online supplemental Figure S7 and S8. Eight studies included in the meta-analysis compared the sensitivity and specificity of ADNEX (with CA125) with those of RMI in surgically managed patients.^34^,^3638^ The summary estimate for sensitivity in the four different meta-analyses varied between 0.92 and 0.94 for ADNEX and was 0.61 for RMI in all four analyses. The summary estimate for specificity varied between 0.76 and 0.85 for ADNEX and between 0.92 and 0.95 for RMI. Specificity was higher for operated and conservatively managed patients combined (ranging from 0.85 to 0.95) than for operated patients (ranging from 0.76 to 0.93). Figure 3 illustrates the centre-specific sensitivities and specificities of ADNEX and RMI in patients managed with surgery in the space of a receiver operating characteristic curve.

**Table 3:** Meta-analysis results for sensitivity and specificity at the 10% risk of malignancy cut-off for ADNEX and at the 200 cut-off for RMI

Meta-analysis	Patients	Studies (centres)	Sensitivity			Specificity		
			Summary est. (95% CI)	95% PI	τ^2^	Summary est. (95% CI)	95% PI	τ^2^
*Target population: operated patients*								
*For ADNEX with CA125*								
ADNEX	5020	8 (26)	0.93 (0.90 to 0.96)	0.71–0.99	0.65	0.77 (0.71 to 0.81)	0.46–0.92	0.38
RMI	5020	8 (26)	0.61 (0.56 to 0.67)	0.38–0.81	0.20	0.92 (0.89 to 0.94)	0.75–0.98	0.39
*For ADNEX without CA125*								
ADNEX	3773	4 (22)	0.94 (0.90 to 0.96)	0.66–0.99	0.81	0.76 (0.70 to 0.81)	0.48–0.91	0.31
RMI	3773	4 (22)	0.61 (0.56 to 0.67)	0.40–0.79	0.16	0.93 (0.90 to 0.95)	0.77–0.98	0.41
*Target population: operated and conservatively managed patients*								
*For ADNEX with CA125*								
ADNEX	4905	1 (17)	0.92 (0.87 to 0.96)	0.55–0.99	0.99	0.85 (0.81 to 0.89)	0.63–0.95	0.30
RMI	4905	1 (17)	0.61 (0.54 to 0.67)	0.38–0.80	0.17	0.95 (0.93 to 0.97)	0.82–0.99	0.44
*For ADNEX without CA125*								
ADNEX	4905	1 (17)	0.92 (0.86 to 0.96)	0.53–0.99	1.07	0.85 (0.8 to 0.88)	0.62–0.95	0.29
RMI	4905	1 (17)	0.61 (0.54 to 0.67)	0.38–0.80	0.17	0.95 (0.93 to 0.97)	0.82–0.99	0.44

ADNEX, Assessment of Different NEoplasias in the adneXa; CI, confidence interval; PI, prediction interval; RMI, risk of malignancy index; t2, τ squared.

The results for the meta-analysis of clinical utility are shown in table 4. Based on data from operated patients, the probability that the model would be clinically useful in a new centre was 95% to 96% for ADNEX and 13% to 15% for RMI. Based on data from both surgically and conservatively managed patients, the probability that the model would be clinically useful in a new centre was 99% for ADNEX and 58% for RMI.

**Table 4:** Meta-analysis for clinical utility of ADNEX and RMI

Meta-analysis	Patients	Studies (centres)	**Net benefit**		**Relative utility**		
			Estimate (95% CrI)	95% PI	Estimate (95% CrI)	95% PI	P (useful)
*Target population: operated patients*							
*For ADNEX with CA125*							
ADNEX	5020	8 (26)	0.29 (0.21 to 0.37)	0.05; 0.70	0.51 (0.40 to 0.61)	−0.11; 0.77	96%
RMI	5020	8 (26)	0.19 (0.13 to 0.27)	0.02; 0.60	−0.80 (−1.3 to −0.37)	−1.30; −0.37	15%
*For ADNEX without CA125*							
ADNEX	3773	4 (22)	0.30 (0.22 to 0.39)	0.07; 0.67	0.48 (0.34 to 0.61)	−0.14; 0.78	95%
RMI	3773	4 (22)	0.20 (0.13 to 0.28)	0.02; 0.58	−0.90 (−1.5 to −0.45)	−3.97; 0.31	13%
*Target population: operated and conservatively managed patients*							
*For ADNEX with CA125*							
ADNEX	4905	1 (17)	0.17 (0.10 to 0.26)	0.02; 0.61	0.69 (0.60 to 0.77)	0.20; 0.85	99%
RMI	4905	1 (17)	0.12 (0.06 to 0.19)	0.01; 0.50	0.05 (−0.27 to 0.32)	−1.58; 0.53	58%
*For ADNEX without CA125*							
ADNEX	4905	1 (17)	0.17 (0.11 to 0.25)	0.02; 0.59	0.68 (0.58 to 0.77)	0.18; 0.85	99%
RMI	4905	1 (17)	0.12 (0.06 to 0.19)	0.01; 0.50	0.05 (−0.27 to 0.32)	−1.58; 0.53	58%

ADNEX, Assessment of Different NEoplasias in the adneXa; RMI, Risk of malignancy index.

### Certainty of evidence

Based on the subdomains of the GRADE assessment, we found that the risk of bias in the studies included in this meta-analysis was a substantial limitation affecting the certainty of our meta-analysis results. Only one study (representing 4905 patients or 59% of the total number of patients included in this review) was not classified as having a high risk of bias.^34^ However, the results of this low-risk of bias study and the current meta-analysis showed consistent findings. Funnel plots for AUC did not suggest publication bias (online supplemental Figure S9).

## Discussion

### Main findings

This meta-analysis indicates that the ability of ADNEX, with or without the inclusion of CA125, outperforms RMI in distinguishing benign from malignant adnexal masses. It also suggests superior clinical utility of ADNEX compared with RMI when applying the commonly recommended cut-offs (10% malignancy risk for ADNEX and RMI score 200) for determining which patients with an adnexal mass should be referred to an oncology centre.

### Strengths and limitations

Our meta-analysis is a comprehensive head-to-head comparison of external validations of ADNEX and RMI. No previous study has conducted a systematic review and thorough meta-analysis comparing these models head-to-head. A limitation is the high risk of bias in all but one of the included studies and the poor reporting of TRIPOD items. Even though this is not a limitation of our meta-analysis, it affects our results. Some of the reasons for high risk of bias, e.g.,unknown or improper handling of missing data (inappropriate exclusions), unclear information on blinding and small sample size may have skewed the results of the included studies, and by extension, the results of our meta-analysis. On the other hand, we found the summary estimates for AUC, sensitivity and specificity in the only study with low risk of bias^34^ (a meta-analysis of data from 17 centres) to be very similar to those of the studies with high risk of bias. There was considerable heterogeneity in model performance across studies. This is probably explained by differences in study populations, settings or study designs. For instance, very heterogeneous populations in terms of the model’s predictors have higher AUCs.^47^ Rather than being a limitation, heterogeneity could be seen as a good representation of how the models perform under different conditions. Performance heterogeneity is commonly observed for prediction models, even in studies that adhere to similar terms and definitions and share a measurement protocol.^34^ Due to the absence of reporting of calibration performance in all but one of the included studies, we could not perform meta-analysis of calibration performance, which is another limitation. It was also not possible to meta-analyse net benefit over a range of risk thresholds.

### Interpretation

There are published meta-analyses that compare the performance of different methods to calculate the risk of malignancy in adnexal masses or to classify adnexal masses as benign or malignant but that do not perform head-to-head comparisons.^48 49^ Meys and colleagues^49^ reported a pooled sensitivity of 0.75 (95% CI 0.72 to 0.79) and a pooled specificity of 0.92 (0.88–0.94) for RMI at the 200 threshold based on 6970 masses from 14 studies. Kaijser and colleagues^48^ found a pooled sensitivity of 0.72 (0.67–0.76) and a pooled specificity of 0.92 (0.89–0.93) based on 5626 patients from 23 studies, that is, their results were similar to those of Meys *et al*.^49^ These studies reported a higher sensitivity than the current study. The most comprehensive meta-analysis of the diagnostic performance of ADNEX (47 studies, 17 007 adnexal masses) reports meta-analysed summary estimates of AUC, sensitivity, specificity and net benefit at the 10% risk of malignancy cut-off.^18^ The results are very similar to those in the current meta-analysis. An older multicentre study (2403 patients; 18 centres) reported a higher clinical utility for ADNEX than for RMI at cut-offs between 25 and 450. However, that study used a preliminary version of the ADNEX model.^50^

In their article published in 1990, Jacobs *et al* discuss the choice of cut-off of RMI to decide which patients to refer to oncological care and state that if the availability of such care is limited, then the cut-off can be set at 75 or 200.^6^ Many guidelines have adopted the 200 cut-off.^7^,^12^ As shown in this meta-analysis, RMI at 200 cut-off has low sensitivity (0.61) but high specificity (≥0.92). At this cut-off, a high proportion of stage one ovarian malignancies, borderline tumours and secondary ovarian metastases will be missed, but most advanced cancers will be detected.^51^ At the 10% risk of malignancy cut-off of ADNEX, recommended in an international consensus statement,^1^ also borderline tumours and stage one primary invasive ovarian malignancies will be detected and therefore referred to an oncology centre, but so will many benign tumours (sensitivity in this meta-analysis≥0.92, specificity≥0.76). This would seem to indicate that lack of gynaecological oncological expertise is no longer a problem (in western countries). This is supported by the recommendation of Sundar *et al*.^12^ Sundar *et al.* did a head-to-head comparison of RMI (cut-off 250) with ADNEX in postmenopausal women with symptoms suggestive of ovarian cancer in a real-world setting (ROCKeTS study). They suggested, based on their results, that IOTA ADNEX at 10% should be considered the new standard-of-care diagnostic in ovarian cancer for postmenopausal patients.^12^ This indicates that today high sensitivity has priority over high specificity. The sensitivity of RMI can be increased by using a lower cut-off, e.g., 25 or 50. However, according to a meta-analysis of data from 23 Italian centres, the clinical utility of ADNEX is superior to that of RMI also at RMI score 25.^52^

## Conclusion

In conclusion, the results of our meta-analysis support that it is time to consider replacing RMI with ADNEX in clinical guidelines, as suggested by Sundar and colleagues.^12^ The need for IOTA certification and adherence to a standardised examination technique and terminology may present a challenge but should be seen as a necessary step in the evolution of modern medical practice. Moreover, ADNEX is integrated into ultrasound machines and calculates the likelihood of four different types of malignant tumours. This makes it a valuable tool for clinicians. ADNEX works well also in the hands of ultrasound examiners with limited ultrasound experience and when used in “a real-world” setting.^12 52^

## Supplementary material

10.1136/bmjopen-2025-104141 online supplemental file 1

## Data Availability

Data are available in a public, open access repository.

## References

[1] Timmerman D, Planchamp F, Bourne T (2021). ESGO/ISUOG/IOTA/ESGE Consensus Statement on preoperative diagnosis of ovarian tumors. Ultrasound Obstet Gynecol.

[2] Ledermann JA, Matias-Guiu X, Amant F (2024). ESGO-ESMO-ESP consensus conference recommendations on ovarian cancer: pathology and molecular biology and early, advanced and recurrent disease. Ann Oncol.

[3] Harter P, Muallem ZM, Buhrmann C (2011). Impact of a structured quality management program on surgical outcome in primary advanced ovarian cancer. Gynecol Oncol.

[4] Aletti GD, Dowdy SC, Gostout BS (2009). Quality improvement in the surgical approach to advanced ovarian cancer: the Mayo Clinic experience. J Am Coll Surg.

[5] Van Calster B, Van Hoorde K, Valentin L (2014). Evaluating the risk of ovarian cancer before surgery using the ADNEX model to differentiate between benign, borderline, early and advanced stage invasive, and secondary metastatic tumours: prospective multicentre diagnostic study. BMJ.

[6] Jacobs I, Oram D, Fairbanks J (1990). A risk of malignancy index incorporating CA 125, ultrasound and menopausal status for the accurate preoperative diagnosis of ovarian cancer. Br J Obstet Gynaecol.

[7] RCOG (2016). Ovarian masses in premenopausal women, management of suspected (green-top guideline no.62). https://www.rcog.org.uk/guidance/browse-all-guidance/green-top-guidelines/ovarian-masses-in-premenopausal-women-management-of-suspected-green-top-guideline-no-62/.

[8] Regionala cancercentrum i samverkan (2023). Äggstockscancer med epitelial histologi.

[9] Staer-Jensen J, Ekerhovd E, Olsen IP Benigne cyster i ovariene.

[10] Poul Bak Thorsen (2016). Håndtering af ovariecyster, opdateret/supplerende guideline 2016.

[11] RCOG (2016). Ovarian cysts in postmenopausal women (green-top guideline no.34). https://www.rcog.org.uk/guidance/browse-all-guidance/green-top-guidelines/ovarian-cysts-in-postmenopausal-women-green-top-guideline-no-34/.

[12] Sundar S, Agarwal R, Davenport C (2024). Risk-prediction models in postmenopausal patients with symptoms of suspected ovarian cancer in the UK (ROCkeTS): a multicentre, prospective diagnostic accuracy study. Lancet Oncol.

[13] (2011). Ovarian cancer: the recognition and initial management of ovarian cancer.

[14] Tingulstad S, Hagen B, Skjeldestad FE (1996). Evaluation of a risk of malignancy index based on serum CA125, ultrasound findings and menopausal status in the pre-operative diagnosis of pelvic masses. Br J Obstet Gynaecol.

[15] Yamamoto Y, Yamada R, Oguri H (2009). Comparison of four malignancy risk indices in the preoperative evaluation of patients with pelvic masses. Eur J Obstet Gynecol Reprod Biol.

[16] Tingulstad S, Hagen B, Skjeldestad FE (1999). The risk-of-malignancy index to evaluate potential ovarian cancers in local hospitals. Obstet Gynecol.

[17] Westwood M, Ramaekers B, Lang S (2018). Risk scores to guide referral decisions for people with suspected ovarian cancer in secondary care: a systematic review and cost-effectiveness analysis. Health Technol Assess.

[18] Barreñada L, Ledger A, Dhiman P (2024). ADNEX risk prediction model for diagnosis of ovarian cancer: systematic review and meta-analysis of external validation studies. *bmjmed*.

[19] Page MJ, McKenzie JE, Bossuyt PM (2021). The PRISMA 2020 statement: an updated guideline for reporting systematic reviews. BMJ.

[20] Snell KIE, Levis B, Damen JAA (2023). Transparent reporting of multivariable prediction models for individual prognosis or diagnosis: checklist for systematic reviews and meta-analyses (TRIPOD-SRMA). BMJ.

[21] Moons KGM, de Groot JAH, Bouwmeester W (2014). Critical appraisal and data extraction for systematic reviews of prediction modelling studies: the CHARMS checklist. PLoS Med.

[22] Collins GS, Reitsma JB, Altman DG (2015). Transparent reporting of a multivariable prediction model for individual prognosis or diagnosis (TRIPOD): the TRIPOD statement. BMJ.

[23] Moons KGM, Wolff RF, Riley RD (2019). PROBAST: A Tool to Assess Risk of Bias and Applicability of Prediction Model Studies: Explanation and Elaboration. Ann Intern Med.

[24] Debray TP, Damen JA, Riley RD (2019). A framework for meta-analysis of prediction model studies with binary and time-to-event outcomes. Stat Methods Med Res.

[25] IntHout J, Ioannidis JPA, Borm GF (2014). The Hartung-Knapp-Sidik-Jonkman method for random effects meta-analysis is straightforward and considerably outperforms the standard DerSimonian-Laird method. BMC Med Res Methodol.

[26] Riley RD, Higgins JPT, Deeks JJ (2011). Interpretation of random effects meta-analyses. BMJ.

[27] Wilson EB (1927). Probable Inference, the Law of Succession, and Statistical Inference. J Am Stat Assoc.

[28] Vickers AJ, Elkin EB (2006). Decision curve analysis: a novel method for evaluating prediction models. Med Decis Making.

[29] Vickers AJ, Van Calster B, Steyerberg EW (2016). Net benefit approaches to the evaluation of prediction models, molecular markers, and diagnostic tests. BMJ.

[30] Wynants L, Riley RD, Timmerman D (2018). Random-effects meta-analysis of the clinical utility of tests and prediction models. Stat Med.

[31] Baker SG (2009). Putting Risk Prediction in Perspective: Relative Utility Curves. JNCI.

[32] Baker SG, Cook NR, Vickers A (2009). Using relative utility curves to evaluate risk prediction. J R Stat Soc Ser A Stat Soc.

[33] Guyatt GH, Oxman AD, Vist GE (2008). GRADE: an emerging consensus on rating quality of evidence and strength of recommendations. BMJ.

[34] Van Calster B, Valentin L, Froyman W (2020). Validation of models to diagnose ovarian cancer in patients managed surgically or conservatively: multicentre cohort study. BMJ.

[35] Díaz L, Santos M, Zambrano B (2017). Ovarian tumors: Risk of malignancy and IOTA ADNEX model indexes. No Technology Doppler Diagnostic Options. Rev Obstet Ginecol Venez.

[36] (2020). Preoperative discriminating performance of the IOTA-ADNEX model and comparison with Risk of Malignancy Index: an external validation in a non-gynecologic oncology tertiary center. *EJGO*.

[37] Behnamfar F, Esmaeilian F, Adibi A (2022). Comparison of Ultrasound and Tumor Marker CA125 in Diagnosis of Adnexal Mass Malignancies. Adv Biomed Res.

[38] Meys EMJ, Jeelof LS, Achten NMJ (2017). Estimating risk of malignancy in adnexal masses: external validation of the ADNEX model and comparison with other frequently used ultrasound methods. Ultrasound Obstet Gynecol.

[39] Qian L, Du Q, Jiang M (2021). Comparison of the Diagnostic Performances of Ultrasound-Based Models for Predicting Malignancy in Patients With Adnexal Masses. Front Oncol.

[40] Sandal K, Polat M, Yassa M (2018). Comparision of “risk of malignancy indices” and “assesment of different neoplasia in the adnexa” (ADNEX) model as preoperative malignancy evaluation methods for adnexal masses. Zeynep Kamil Tip Bul.

[41] Szubert S, Szpurek D, Wójtowicz A (2020). Performance of Selected Models for Predicting Malignancy in Ovarian Tumors in Relation to the Degree of Diagnostic Uncertainty by Subjective Assessment With Ultrasound. J Ultrasound Med.

[42] Wang R, Yang Z (2023). Evaluating the risk of malignancy in adnexal masses: validation of O-RADS and comparison with ADNEX model, SA, and RMI. *Ginekol Pol*.

[43] Poonyakanok V, Tanmahasamut P, Jaishuen A (2023). Prospective comparative trial comparing O-RADS, IOTA ADNEX model, and RMI score for preoperative evaluation of adnexal masses for prediction of ovarian cancer. J Obstet Gynaecol Res.

[44] Borges AL, Brito M, Ambrósio P (2024). Prospective external validation of IOTA methods for classifying adnexal masses and retrospective assessment of two‐step strategy using benign descriptors and ADNEX model: Portuguese multicenter study. Ultrasound in Obstet &Amp; Gyne.

[45] Oun RDA, Hamzah HJ, Salman AH (2023). Preoperative risk assessment tests for suspicious ovarian mass. Onkol Radioter.

[46] Vilendecic Z, Radojevic M, Stefanovic K (2023). Accuracy of IOTA Simple Rules, IOTA ADNEX Model, RMI, and Subjective Assessment for Preoperative Adnexal Mass Evaluation: The Experience of a Tertiary Care Referral Hospital. Gynecol Obstet Invest.

[47] van Leeuwen FD, Steyerberg EW, van Klaveren D (2025). Instability of the AUROC of Clinical Prediction Models. Stat Med.

[48] Kaijser J, Sayasneh A, Van Hoorde K (2014). Presurgical diagnosis of adnexal tumours using mathematical models and scoring systems: a systematic review and meta-analysis. Hum Reprod Update.

[49] Meys EMJ, Kaijser J, Kruitwagen R (2016). Subjective assessment versus ultrasound models to diagnose ovarian cancer: A systematic review and meta-analysis. Eur J Cancer.

[50] Wynants L, Timmerman D, Verbakel JY (2017). Clinical Utility of Risk Models to Refer Patients with Adnexal Masses to Specialized Oncology Care: Multicenter External Validation Using Decision Curve Analysis. Clin Cancer Res.

[51] Van Holsbeke C, Van Calster B, Bourne T (2012). External validation of diagnostic models to estimate the risk of malignancy in adnexal masses. Clin Cancer Res.

[52] Moro F, Momi M, Ledger A (2025). External validation of ultrasound-based models for differentiating between benign and malignant adnexal masses: a nationwide prospective multicenter study (IOTA phase 6). American Journal of Obstetrics & Gynecology.

[53] Barreñada L, Ledger A, Collins G (2024). ADNEX vs RMI: Head to head comparison.

