# Supplementary material for: Head-to-head comparison of the RMI and ADNEX models to estimate the risk of ovarian malignancy: a systematic review and meta-analysis of external validation studies
Source: BMJ Open. 2025 Oct 7;15(10):e104141. doi: 10.1136/bmjopen-2025-104141 (PMC12506209; doi:10.1136/bmjopen-2025-104141)
Supplement: Supplementary file 2 [file bmjopen-2025-104141.reviewer_comments.pdf]

PEER REVIEW HISTORY

BMJ Open publishes all reviews undertaken for accepted manuscripts. Reviewers are asked to complete a checklist review form and are provided with free text boxes to elaborate on their assessment. These free text comments are reproduced below.

ARTICLE DETAILS

Title (Provisional)

Head-to-head comparison of the RMI and ADNEX models to estimate the risk of ovarian malignancy: a systematic review and meta-analysis of external validation studies

Authors

Barreñada, Lasai; Ledger, Ashleigh; KOTLARZ, Agnieszka; Dhiman, Paula; Collins, Gary Stephen; Wynants, Laure; Verbakel, Jan Yvan Jos; Valentin, Lil; Timmerman, Dirk; Van Calster, Ben

---

VERSION 1 - REVIEW

**Reviewer**	**1**
**Name**	**Dack, Kyle**
**Affiliation**	**University of Bristol, MRC Integrative Epidemiology Unit**
**Date**	**18-Jun-2025**
**COI**	**None**

Statistical review summary:

The comparison uses valid methods. However, there are some important elements missing such as certainty of evidence evaluation, a summary of the studies, and an exploration of heterogeneity. I have also made suggestions to strengthen the clarity of the results by referencing specific statistics more often.

General:

1. There are references to supplementary materials in the text, but these were missing from the files which were available to me.

Abstract:

2. Line 3. I suggest adding the full meaning of the two acronyms introduced on this line.

3. Line 15. I think it would be easier for readers to place the model before the result, e.g. “the summary AUC to distinguish benign from malignant tumours in operated patients for ADNEX was 0.92 (95% CI 0.90-0.94)”.

4. It may be hard for readers to understand from solely the abstract what is meant by clinical utility in terms of how this can be represented as a percentage, suggest clarifying in simple terms (e.g. probability the model is useful in a new centre).

Introduction:

5. Very clear and well written, no suggestions.

Methods:

6. The PRISMA item "certainty of evidence" references the statistical analysis. However, I could not find anything which seemed specific to evaluating the certainty of evidence in this section. The results also do not comment on the evidence certainty. I would suggest the recent Core GRADE framework published in the BMJ: https://www.bmj.com/content/389/bmj-2024-081903 which summarises the overall strength of evidence.

7. Statistical analysis: what was the rationale for selecting a random effects over fixed effects model? I suggest adding this to give more transparency to the statistical decision making.

8. "We compared ADNEX with CA125 with RMI and ADNEX without CA125 with RMI in two populations" this is quite hard to tell what the two comparisons are, I suggest using commas or numberings to clarify.

Results

9. I am unsure if a flow chart was produced or not, it may be Figure S1 but this was not in the files made available to me.

10. "the summary estimate for the AUC varied between 0.92 and 0.94 for ADNEX, and between 0.85 and 0.89 for RMI" it is not clear to me which statistics these are referring to, because it does not correspond to the 95% CI or 95% PI.

11. In addition to reporting the summary estimate I also suggest commenting on the heterogeneity of each meta-analysis. Readers may be unfamiliar with interpreting tau squared and 95% PI measures of heterogeneity which are less commonly reported than I2, so in-text description would be very valuable in helping readers to interpret. My interpretation would be that this reflects a moderate amount of heterogeneity, and it is notable that the 95% PI of the two models overlaps (although they do not overlap in terms of 95% CI).

12. "The AUC was higher for operated and conservatively managed patients combined than for operated patients only." To aid readers, it would help to report the specific statistics/ranges in-text. This suggestion applies to a much of the results, because otherwise readers must swap between reading the text and tables often.

13. Table 1 provides aggregated statistics of study characteristics which I find helpful. However, a standard summary table describing each study individually should also be

included. Currently there is no single table listing all the studies included, their populations, methods, etc.

Discussion

14. The validity of the meta-analysis applying to all populations depends on the level of heterogeneity. This is not really commented on, or what the authors consider to be sources of the heterogeneity. Subgroup analysis was not performed which is reasonable given the small number of studies, but a qualitative exploration of potential causes could still be provided in the discussion.

**Reviewer**	**2**
**Name**	**Abramowicz, Jacques**
**Affiliation**	**University of Chicago**
**Date**	**23-Jun-2025**
**COI**	**None**

In the manuscript bmjopen-2025-104141, "Head-to-head comparison of the RMI and ADNEX models to estimate the risk of ovarian malignancy: a systematic review and meta-analysis of external validation studies" the authors performed a head to-head comparisons between the ADNEX model (with and without CA125) and the RMI with a systematic literature search that yielded 11 studies with 8271 tumors. Their conclusion was that the ADNEX model has better discrimination with a summary AUC of 0.92 in operated patient versus 0.85 for RMI. Sensitivity and specificity were 0.93 and 0.77 respectively for the ADNEX model and 0.61 and 0.93 for RMI. This is an excellent article with remarkable description of the methodology.

Title: appropriate

Abstract: adequate

Background: appropriate

Eligibility criteria: lines 20-23: sentence is incomplete

Results: well described

Discussion: appropriate with adequate citation of the published literature.

References: why is there a * in reference #2? References 8, 9, 10 cannot be accessed.

Figures and tables: appropriate.

VERSION 1 - AUTHOR RESPONSE

REVIEWER 1 COMMENT<br>Dr. Kyle Dack	AUTHOR RESPONSE

**1. There are references to supplementary materials in the text, but these were missing from the files which were available to me.**	Due to some technical reasons the Supplementary material was not uploaded but now you should be able to access it.
**Abstract:**<br>**2. Line 3. I suggest adding the full meaning of the two acronyms introduced on this line.**	Thanks for the suggestions. We added now in full.
**3. Line 15. I think it would be easier for readers to place the model before the result, e.g. "the summary AUC to distinguish benign from malignant tumours in operated patients for ADNEX was 0.92 (95% CI 0.90-0.94)".**	Thank you for the comment. We agree and have now placed the model before reporting the metric both for AUC and Sen/Spe
**4. It may be hard for readers to understand from solely the abstract what is meant by clinical utility in terms of how this can be represented as a percentage, suggest clarifying in simple terms (e.g. probability the model is useful in a new centre).**	Thank you, we agree. We now clarify what encompasses clinical utility.
**Methods:**<br>**6. The PRISMA item "certainty of evidence" references the statistical analysis. However, I could not find anything which seemed specific to evaluating the certainty of evidence in this section. The results also do not comment on the evidence certainty. I would suggest the recent Core GRADE framework published in the BMJ: https://www.bmj.com/content/389/bmj-2024-081903 which summarises the overall strength of evidence.**	Thank you for the suggestion. We updated the manuscript and write that we used an adapted version of GRADE as in the systematic review of ADNEX. We now state in the methods section:<br>*Reporting bias and small study effects were visually explored with funnel plots adapted for the AUC. The body of evidence was assessed with an adapted version of GRADE (Grading of Recommendations Assessment, Development and Evaluation) (33)*<br><br>And in the results section we added a subsection of "Certainty of evidence" stating:<br>*Based on the subdomains of the GRADE assessment, we found that the risk of bias in the studies included in this meta-analysis was a substantial limitation affecting the certainty of our meta-analysis results. Only one study (representing 4905 patients or 59% of the total patients included in this review) was not classified as having a high risk of bias (34). However, the results of this low-risk of bias study and the current meta-analysis showed consistent findings. Funnel plots for AUC did not suggest publication bias (Figure S9)*<br>We also included Figure S9 as a funnel plot adapted for the AUC of ADNEX and RMI.
**7. Statistical analysis: what was the rationale for selecting a random effects over fixed effects model? I suggest adding this to give**	Thank you for the comment. We now clarified by adding:<br>*We use random effects model due to the anticipated between study heterogeneity due to*

**more transparency to the statistical decision making.**	*different populations, settings or study designs (24)*
**8. "We compared ADNEX with CA125 with RMI and ADNEX without CA125 with RMI in two populations" this is quite hard to tell what the two comparisons are, I suggest using commas or numberings to clarify.**	Thank you for the suggestions. We clarified that paragraph which now reads: *We compared ADNEX with CA125 vs RMI, and ADNEX without CA125 vs RMI in two populations: 1) only operated patients, and 2) operated and conservatively managed patients combined. Thus, four meta-analyses were performed: 1) ADNEX with CA125 vs RMI in operated patients, 2) ADNEX without CA125 vs RMI in operated patients, 3) ADNEX with CA125 vs RMI in operated and conservatively managed patients combined, and 4) ADNEX without CA125 vs RMI in operated and conservatively managed patients combined.*
**Results**<br>**9. I am unsure if a flow chart was produced or not, it may be Figure S1 but this was not in the files made available to me.**	This should be now available in the uploaded Supplementary material.
**10. "the summary estimate for the AUC varied between 0.92 and 0.94 for ADNEX, and between 0.85 and 0.89 for RMI" it is not clear to me which statistics these are referring to, because it does not correspond to the 95% CI or 95% PI.**	This is the summary estimates across the different meta-analyses that we performed (i.e. ADNEX with CA125 vs RMI in operated, ADNEX without CA125 vs RMI in operated, ADNEX with CA125 vs RMI in all, ADNEX without CA125 vs RMI in all). We clarified now by adding: The summary estimate for the AUC **in the four different meta-analyses** varied between
**11. In addition to reporting the summary estimate I also suggest commenting on the heterogeneity of each meta-analysis. Readers may be unfamiliar with interpreting tau squared and 95% PI measures of heterogeneity which are less commonly reported than I2, so in-text description would be very valuable in helping readers to interpret. My interpretation would be that this reflects a moderate amount of heterogeneity, and it is notable that the 95% PI of the two models overlaps (although they do not overlap in terms of 95% CI).**	Thank you for the comment. Just some clarifications, I2 presents the proportion of total variability due to between study heterogeneity. It has been shown to not be an easy measure of heterogeneity to interpret because it increases as sample sizes increases while tau squared (the variance due to between-study heterogeneity) is constant. For that reason, we simply present PI and tau squared. We have added in the methods section *"Tau squared is the between-centre variance in the logit AUC, sensitivity or specificity, with higher values indicating more heterogeneity. The 95% prediction interval reflects the range of AUC, sensitivity or specificity we might expect in new centres: the wider, the larger the between-centre differences in performance.*" The forest plots (Figs S5-S8) show the heterogeneity in a visual way and Tables 2-3 in a numerical way. There are no rules of thumb of what a wide PI is, this is context-dependent.

**12. "The AUC was higher for operated and conservatively managed patients combined than for operated patients only." To aid readers, it would help to report the specific statistics/ranges in-text. This suggestion applies to a much of the results, because otherwise readers must swap between reading the text and tables often.**	Thank you for the suggestion. We now included the ranges in parentheses.
**13. Table 1 provides aggregated statistics of study characteristics which I find helpful. However, a standard summary table describing each study individually should also be included. Currently there is no single table listing all the studies included, their populations, methods, etc.**	We fully agree. Such tables were part of the supplementary material not uploaded due to the technical error mentioned above. You can find the information in Tables S3 and S4.
**Discussion**<br>**14. The validity of the meta-analysis applying to all populations depends on the level of heterogeneity. This is not really commented on, or what the authors consider to be sources of the heterogeneity. Subgroup analysis was not performed which is reasonable given the small number of studies, but a qualitative exploration of potential causes could still be provided in the discussion.**	Thank you for the comment. The key of using random effects meta-analysis and presenting prediction intervals is that intervals will account for between study heterogeneity. The summary estimate of each metric is the average across studies, but the expected performance in a new centre is represented by the prediction interval. Following the suggestion we now include in Strengths and limitations:<br>*There was considerable heterogeneity in performance between studies. This is probably explained by differences in study populations, settings or study designs. For instance, very heterogeneous populations in terms of the model's predictors have higher AUCs (47). Rather than being a limitation heterogeneity could be seen as a representation of how the models perform under different conditions. Performance heterogeneity is commonly observed for prediction models, even in studies that adhere to same terms and definitions and share a measurement protocol, such as multicenter studies (34).*

REVIEWER 2 COMMENT<br>Prof. Jacques Abramowicz	AUTHOR RESPONSE
**Eligibility criteria: lines 20-23: sentence is incomplete**	Even if the sentence might look incomplete this is a continuation of the enumeration of exclusion criteria .
**References: why is there a * in reference #2? References 8, 9, 10 cannot be accessed.**	Thank you for spotting the error in reference 2, for references 8-10 we could retrieve the documents from the available URL
